# Comparison of the Classification Results Accuracy for CT Soft Tissue and Bone Reconstructions in Detecting the Porosity of a Spongy Tissue

**DOI:** 10.3390/jcm11154526

**Published:** 2022-08-03

**Authors:** Róża Dzierżak, Zbigniew Omiotek, Ewaryst Tkacz, Sebastian Uhlig

**Affiliations:** 1 Department of Electronics and Information Technology, Lublin University of Technology, ul. Nadbystrzycka 38 A, 20-618 Lublin, Poland; z.omiotek@pollub.pl; 2 Department of Biosensors and Processing of Biomedical Signals, Silesian University of Technology, ul. Roosevelta 40, 44-800 Zabrze, Poland; etkacz@polsl.pl; 3 Department of Medical Radiology, Medical University of Lublin, ul. Jaczewskiego 8, 20-090 Lublin, Poland; sebastian.uhlig@icloud.com

**Keywords:** osteoporosis, soft tissue reconstruction, bone reconstruction, texture analysis, classification

## Abstract

The aim of the study was to compare the accuracy of the classification pertaining to the results of two types of soft tissue and bone reconstructions of the spinal CT in detecting the porosity of L1 vertebral body spongy tissue. The dataset for each type of reconstruction (high-resolution bone reconstruction and soft tissue reconstruction) included 400 sponge tissue images from 50 healthy patients and 50 patients with osteoporosis. Texture feature descriptors were calculated based on the statistical analysis of the grey image histogram, autoregression model, and wavelet transform. The data dimensional reduction was applied by feature selection using nine methods representing various approaches (filter, wrapper, and embedded methods). Eleven methods were used to build the classifier models. In the learning process, hyperparametric optimization based on the grid search method was applied. On this basis, the most effective model and the optimal subset of features for each selection method used were determined. In the case of bone reconstruction images, four models achieved a maximum accuracy of 92%, one of which had the highest sensitivity of 95%, with a specificity of 89%. For soft tissue reconstruction images, five models achieved the highest testing accuracy of 95%, whereas the other quality indices (TPR and TNR) were also equal to 95%. The research showed that the images derived from soft tissue reconstruction allow for obtaining more accurate values of texture parameters, which increases the accuracy of the classification and offers better possibilities for diagnosing osteoporosis.

## 1. Introduction

A CT image is obtained through transformations and mathematical calculations performed during the measurement process. They are based on an attempt to recreate the damping of a radiation beam in an object through a series of measurements. The process of transforming the primary data into a CT image is known as reconstruction. In order to form a CT image, a computer system assigns a single value in Hounsfield (HU) scale to each pixel. The obtained radiodensity values are a mean from the weakening of radiation beams going through a given point [1,2,3]. Due to the diversity of the structures in the human body, it is necessary to employ various reconstruction filters. While assessing the condition of organs with a high contrast of internal structures, e.g., bones or lungs, the high-resolution bone reconstruction (“hard kernel” algorithm) is carried out, which enhances the edge quality. In the assessment of soft tissues or organs with lower contrast of internal structures, the soft tissue reconstruction (“soft kernel” algorithm) is employed, which reduces noise at the expense of lowering the spatial resolution. Owing to the application of various reconstruction filters, adjustment of the image for further analyses is possible [4,5].

One of the methods for determining the condition of the analyzed tissue is texture analysis [6]. Although this method can be successfully used in many diagnostic processes, new applications are still being sought, which is indicated by numerous scientific publications [7,8,9,10,11,12,13,14,15,16,17,18]. This paper presented the process for the classification of spongy matter images in the L1 lumbar vertebra on the basis of the obtained texture properties. The spongy matter is considered bone tissue; however, their lamellae are arranged loosely, and their mineralization level is approximately a third of that in compact bone. Decreasing the mineral density of the spongy matter leads to porosis, initially in the form of osteopenia and then osteoporosis [19,20,21]. Dual-energy X-ray Absorptiometry (DXA), based on the measurement of bone density, is a commonly employed method for diagnosing osteoporosis. The mineral content in bones governs the number of minerals in the measurement spot, which is divided by the surface, yielding bone mineral density (BMD). Therefore, this technique provides information on the mineral density of the entire examined region without the precise determination of the segment with deficiencies. The index T corresponding to the standard deviation from the reference mineral density is used to classify a patient as healthy or ill. The T = −1 value is considered to be normal; ranges of −1 to 2.4 corresponds to osteopenia, while −2.5 and less are recognized as osteoporosis. Reduced bone mineral density (BMD) identified through DXA requires differential diagnosis to determine its causes. The blood tests and urinalyses are conducted for this purpose [22,23,24].

The course of osteoporosis is symptomless in its early stages. It is usually diagnosed in the advanced stage, when osteoporotic fractures may occur, even without injuries. Therefore, preventive examinations, aiming at detecting the condition at an early stage and mitigating its consequences, are essential [22,24]. For this reason, new diagnostic solutions enabling the diagnosis of osteoporosis at an early stage are being sought. The literature contains descriptions of numerous experiments connected to seeking methods for identifying lesions in bone regions. Mustapha et al. [25,26] and Stanley et al. [27] presented the method for identifying lesions in neck vertebrae. Mustapha et al. [25] describe a method for AOs (classes and severity) classification of cervical radiography by designing a fuzzy decision tree (FDT) model. The results obtained on a set of 400 cervical vertebrae radiography images indicate the classification rate of 93.09%. Stanley et al. [27], based on the size invariant descriptor (SID), *K*-means (Km), and nearest neighbor methods (NN), the obtained classification rate reached 84.44%. In turn, Mustapha et al. in [26] the employing region-based (RB) fracture characterization as well as five-fold cross-validation (5FCV), and an efficiency of 87.58% was achieved. Lespessailles et al. [28] show clinical interest in bone texture analysis with a new high-resolution X-ray device. The studies indicated that the combination of BMD and texture parameter values provided a better assessment of the fracture risk than that obtainable solely by BMD measurement. The texture analysis also found an application in the diagnostic of pelvic bones, which was described in [29] by Gaidel et al. It was demonstrated that the covariance features were the most efficient—the diagnostic error probability of 0.2 was obtained. Similar findings were also presented in Chappard et al. [30] and Lespessailles et al. [31]. These works also demonstrated the relationship between BMD and texture parameters.

During the assessment of the spongy matter, both by radiologists during the standard diagnostic assessment and in the majority of papers on this subject [32,33], the images from bone reconstruction are used. Due to the atypical character of the spongy matter, which was mentioned above, two types of image reconstruction were employed, i.e., soft and hard kernel reconstruction. The main aim of the study was to verify which type or reconstructed images enable obtaining the texture and allow for a more precise classification of the tissue condition.

## 2. Materials and Methods

### 2.1. Material

The imaging examinations considered in the paper were carried out in a hospital (Samodzielny Szpital Kliniczny nr 4) in Lublin. Patients were referred to the lumbosacral spine examination from an orthopaedic clinic and ER. The examination involved the analysis of the lumbosacral spine CT of 100 patients. The control group comprised 50 individuals without the symptoms of osteoporosis or osteopenia. The same number of patients constituted the group of people with osteoporosis. The control group included 26 women and 24 men aged 53 to 77. Each of the patients was examined by means of 32-row CT by GE in a standard protocol for lumbosacral spine examinations. The examination was performed in the spiral acquisition and assessed using multi-surface and three-dimensional reconstructions. The layers of the soft kernel and hard kernel reconstructions corresponded were 2.5 mm and 1.25 mm, respectively. The exposure parameters were adjusted in the range from 85 mA to 700 mA (median = 181) for the intensity and two values—120 kV or 140 kV in the case of voltage.

The assignment of patients to both groups was performed on the basis of the radiology report and the measurement of radiological densities of the spongy matter of the first vertebra of the lumbar spine (L1). On the basis of the literature [34], the border value of tissue density of 120 Hounsfield Units (HU) was assumed. The patients in whom the density was greater than the limit value and the report did not indicate lesions in the spongy matter were assigned to the control group (HEALTHY class). In turn, the patients who did not meet the criteria were assigned to the osteoporotic group (OSTEOPOROSIS class).

### 2.2. Image Preprocessing

**Figure 1:**
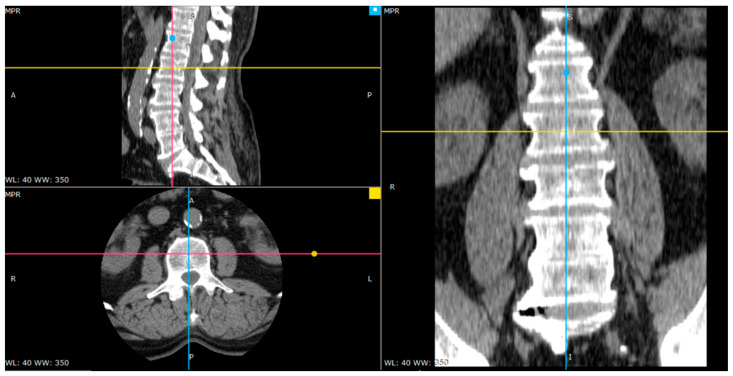
The arrangement of the axis in the center of one of the vertebrae (image in three projections).

The source data saved in the DICOM standard contained RGB images in the 512 × 512 resolution. The images which show the interior of the vertebra along with the spongy matter were selected (Figure 1).

**Figure 2:**
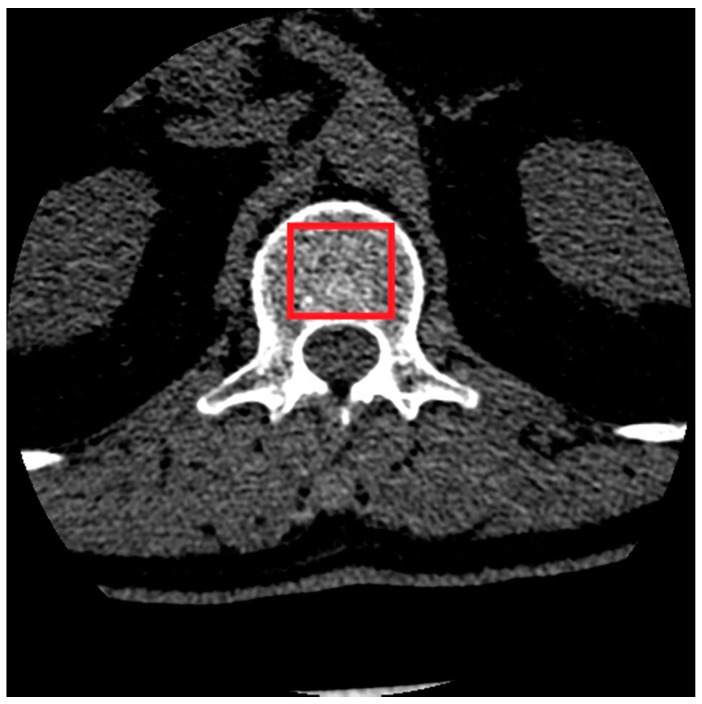
Manual selection of the spongy matter region.

**Figure 3:**
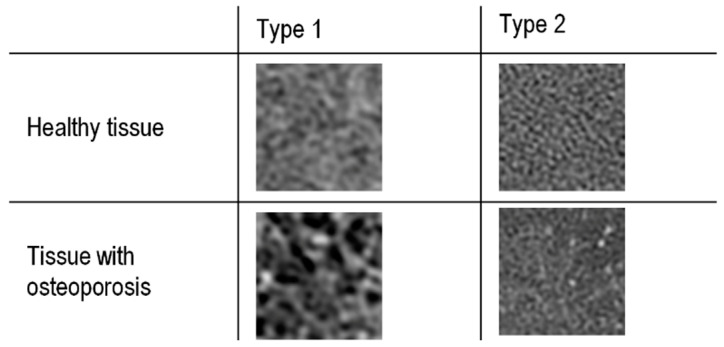
Spongy matter structure in soft-tissue (Type 1) and bone reconstruction (Type 2).

The images on the same vertebra level were selected for soft tissue and bone reconstruction. Since the thickness of layers for bone reconstruction was halved, every other layer of a given vertebra cross-section was analyzed. The images selected for further studies were saved in BMP format and converted from 24-bit RGB to 8-bit greyscale. The extraction of the regions of interest (ROI) was performed manually (Figure 2). The size of the extracted samples was selected to use the textured surface, potentially containing the information in the image of the transverse vertebra section, to the maximum extent. This resulted in samples with a size of 50 × 50 pixels (Figure 3).

During the preprocessing, the image histogram normalization process was omitted because the available results indicate that this operation deteriorates the classification accuracy in the range from 4% for the TPR coefficient (classification sensitivity) to 14% for ACC (overall classification accuracy) [35].

### 2.3. Estimation of Texture Parameters

The prepared samples were subjected to tissue image texture analysis. The image analysis was conducted using the MaZda software (version 4.6) developed in the Institute of Electronics of Lodz University of Technology (Lodz, Poland) [36]. This software was made available online for free for scientific purposes. It enables the analysis of greyscale texture images and indicates the numerical values of the image features. A detailed description of these features can be found in [37,38,39,40], as well as in the MaZda manual. The advantage of this software is the fact that apart from the statistical approach to the image analysis, it also employs a mathematical model (autoregression model) and a transformation approach (wavelet transform).

The set of features has been obtained on the basis of:

- Histogram (9 features): histogram mean, histogram variance, histogram skewness, histogram kurtosis, percentiles 1%, 10%, 50%, 90%, and 99%;
- Gradient (5 features): absolute gradient mean, absolute gradient variance, absolute gradient skewness, absolute gradient kurtosis, percentage of pixels with a nonzero gradient;
- Run length matrix (5 features × 4 various directions): run length nonuniformity, grey level nonuniformity, long run emphasis, short run emphasis, the fraction of image in runs;
- Co-occurrence matrix (11 features × 4 various directions × 5 between-pixels distances) angular second moment, contrast, correlation, sum of squares, inverse difference moment, sum average, sum variance, sum entropy, entropy, difference variance, difference entropy;
- Autoregressive model (5 features): parameters Θ1, Θ2, Θ3, Θ4, standard deviation.
- Haar wavelet (24 features): wavelet energy (features are computed at 6 scales within 4 frequency bands LL, LH, HL, and HH).

The available research results show that the textural features obtained with the above-mentioned methods allowed for high classification accuracy of the ultrasound and radiographic images [41,42].

### 2.4. Data Preprocessing

Two sets of observations were used in the studies; the first contained the images produced through bone reconstruction, while the other used those from soft-tissue reconstruction. For both types of reconstruction, a full set comprised 400 observations, 200 per class. At the beginning of preprocessing, both sets were divided randomly into learning and testing sets. The division was performed in such a way that 70% constituted the learning set and 30%—the test set. As a result, the learning set comprised 280 observations (137 healthy cases and 143 osteoporosis cases), while the test set involved 120 observations (63 healthy cases and 57 osteoporosis cases).

Due to the fact that the feature descriptors were measured at different scales (interval scale, ratio scale), the features were scaled through standardization [43]:

$$z_{ij}=(x_{ij}-{\bar{x}}_{j})/s_{j},$$

where *x_ij_*—value of feature *j* for observation *i*; *z_ij_*—standardized value for feature *j* for observation *i*; ${\bar{x}}_{j}$—arithmetical mean for feature *j*; *s_j_*—standard deviation for feature *j*. Following standardization, the interval measurement scale and normal distribution N(0, 1) were used for all features. Scaling was performed once for the learning data. Then, the test sets were transformed in a similar manner. The vectors of mean values and variance obtained during the standardization of learning sets were used for each feature.

In the following step, the four-stage data cleansing procedure was carried out:

1. Removal of features with constant values (variance equal to 0).
2. Removal of features with nearly constant values (variance lower than 0.01).
3. Removal of duplicated features.
4. Removal of correlated features

The Pearson correlation coefficient (r), which detects linear dependencies and assumes the normality of their distribution, was employed. The features for which |r| > 0.9—indicating strong correlation—were removed.

As a result of data cleansing, the number of features was reduced from 290 to 32 for the bone reconstruction and from 290 to 18 for the soft-tissue reconstruction. The data preprocessing was carried out with the *scikit-learn* library and Python programming language, which was also employed in the further stages of the study.

### 2.5. Data Reduction

The study involved the reduction of the data through feature selection. Generally, the aim of the selection is to limit the initial (complete) set of features to a certain subset, containing the features that are important from the point of view of the applied criterion. In the course of the data cleansing process, the complete set of 290 features was greatly reduced—to 32 for bone reconstruction and 18 for soft-tissue reconstruction. This mainly had an influence on the occurrence of features with a strong correlation (|r| > 0.9). Therefore, the additional reduction was omitted, and the creation of a ranking list by each of the employed methods was set as a goal. The ranking list contained a set of features in the order reflecting their significance in terms of discrimination of observations belonging to different classes. The choice of an optimal number of features for each of the employed selection methods was made in the following stage, which involved training the classification model. In order to avoid overtraining the classifiers, the selection of features was carried out only in relation to the training sets. Then, its results were employed for the transformation of test sets. The selection of features was performed with *scikit-learn*, *scikit-feature*, *ReliefF*, *MLxtend*, and *LightGBM* libraries.

Nine methods belonging to the three following groups were used:

1. Filter methods:
  - Univariate—Fisher’s method (FISHER) and variance analysis method (ANOVA);
  - Multivariate—Relief method (RELIEF).
2. Wrapper methods:
  - Sequential Forward Selection (SFS);
  - Sequential Backward Selection (SBS);
  - Recursive Feature Elimination along with LogisticRegression estimator (RFE).
3. Embedded methods:
  - SelectFromModel meta-transformer and logistic regression estimator (LR);
  - SelectFromModel meta-transformer and AdaBoost estimator (ADA);
  - SelectFromModel meta-transformer along with LightGBM estimator (LGBM).

### 2.6. Training the Classification Models

The training of the classification models aimed to obtain the most efficient model as well as an optimal subset of features for each of the employed selection methods. In the course of this process, the number of features of the learning set changed in accordance with the ranking list. For bone reconstruction, the number of features changed from 2 to 32, except for the methods employing the logistic regression model and AdaBoost model, for which the number of features changed from 2 to 12. In the case of soft-tissue reconstruction, the number of features changed from 2 to 18. The exceptions included the methods based on the logistic regression model and AdaBoost model, for which the number of features changed from 2 to 5 and from 2 to 9, respectively. In the training process, optimal hyperparameter values for a given model were determined in accordance with the grid search method. The GridSearchCV method was employed for this purpose, available in the model_selection module of the scikit-learn library. The model assessment was carried out on the basis of 10-fold cross-validation. Each model considered optimal for a given number of features was saved as a file on the disc. Then, the best model, i.e., the one which ensured the highest classification accuracy at a minimum number of features, was selected from this set. The selection of the best model enabled the unequivocal determination of the optimal set of features that were applied by that model. The above-mentioned procedure was employed for all filter and wrapper methods. In the case of the embedded methods, only the classifiers employed in the selection process were built and adjusted.

Eleven classification methods were used for the features selected by means of the filter and wrapper methods. They were: linear and quadratic discriminant analysis (LDA, QDA), gaussian naive Bayes (BAYES), support vector machines (SVM) that uses regularization parameter C), support vector machines (NuSVM) that uses a parameter to control the number of support vectors nu), *K*-nearest neighbors (KNN), decision tree (DT), multi-layer perceptron (MLP) and three ensemble methods—random forest (RF), gradient boosting (GRAD), AdaBoost (ADA). In turn, three classification methods have been used for the features selected by means of the embedded methods: AdaBoost, logistic regression (LR), and LightGBM (LGBM). All algorithms of the applied machine learning methods (except LightGBM) are implemented in the scikit-learn library.

**Figure 4:**
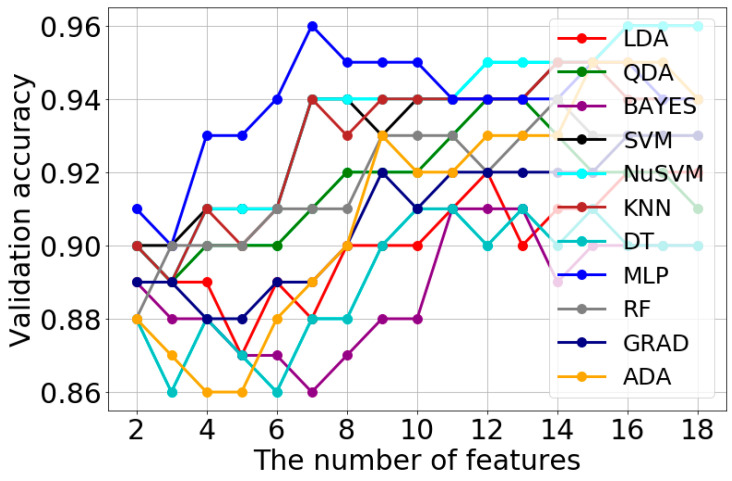
Exemplary process of the model learning and validation for a set of features obtained with the Fisher method (soft-tissue reconstruction) for different classification methods. The graph shows that—for instance—the optimal number of features for the MLP model is 7.

Figure 4 presents an exemplary course of model learning and validation for the set of features obtained with the Fisher method for soft-tissue reconstruction. In turn, Table 1 shows the exemplary results of hyperparametric optimization with the grid search method for the Fisher method and the same type of reconstruction.

The exemplary results in Table 1 indicate that the highest validation accuracy (0.96) is provided by the SVM, NuSVM, and MLP models. However, due to the lowest number of the training set features (7), the MLP model was assumed as the best for the set of features obtained with the Fisher method (grey background).

## 3. Results

Table 2 shows the complete results pertaining to the selection of the optimal models for all feature selection methods and both types of reconstruction. The information in the table contains the symbols of the employed classification method and the selection method applied for building the feature ranking, as well as the number of features used for model construction and its validation accuracy.

**Figure 5:**
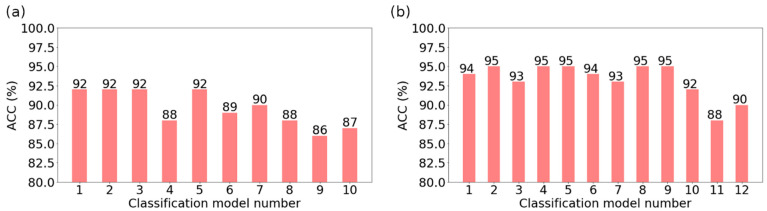
Results of testing the models considered optimal for particular feature selection methods: (**a**) bone reconstruction; (**b**) soft tissue reconstruction.

The evaluation of the optimal methods was carried out through independent test sets. As a result, the most efficient selection method, optimal feature subset indicated with this method, and the best classifiers were obtained. The applied classification quality measures included overall accuracy (ACC), true positive rate (TPR), and true negative rate (TNR). For bone reconstruction (Figure 5a), the highest accuracy (ACC = 92%) was obtained by models no. 1, 2, 3, and 5 (Table 2), at the same validation accuracy of 94%. In the case of the soft-tissue reconstruction (Figure 5b), the highest accuracy (ACC = 95%) was obtained for models no. 2, 4, 5, 8, and 9 (Table 2). The validation accuracy for these models amounted to 96%.

**Figure 6:**
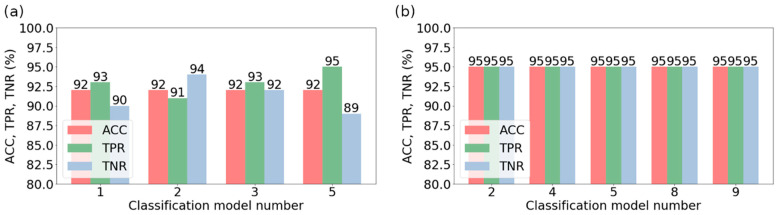
The sensitivity (*TPR*) and specificity (*TNR*) of the models that have reached the highest value of the overall classification accuracy (*ACC*): (**a**) bone reconstruction; (**b**) soft-tissue reconstruction.

**Figure 7:**
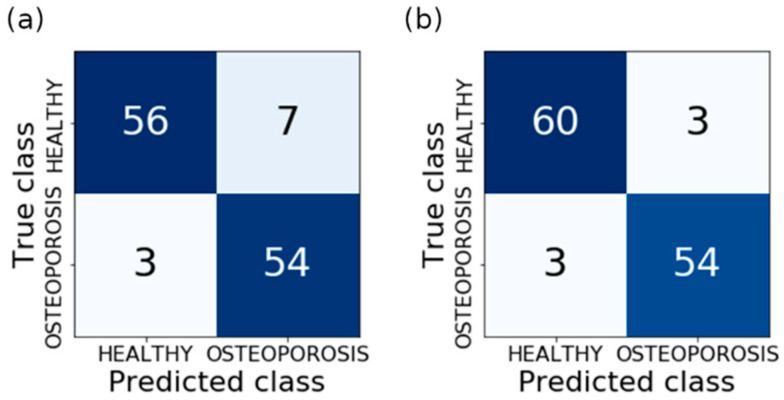
Confusion matrices: (**a**) model 5 for bone reconstruction; (**b**) models 2, 4, 5, 8 and 9 for soft tissue reconstruction.

Figure 6 shows more information on the model evaluation results. Apart from the total classification accuracy, it also presents the true positive rate (sensitivity) and true negative rate (specificity). In the case of bone reconstruction, Figure 6a shows the value of TPR and TNR for models no. 1, 2, 3, and 5, for which ACC = 92%. In turn, for the soft-tissue reconstruction (Figure 6b), the information on TPR and TNR pertains to models no. 2, 4, 5, 8, and 9, which achieved ACC = 95%. In the case of the same ACC value, the TPR value was assumed as the criterion for the selection of the best model due to the medical benefits of such an approach. Therefore, model no. 5 turned out to be the most efficient in bone reconstruction, achieving a TPR of 95%. This model was built with the *K*-nearest neighbors method (for *K* = 1), employing nine features obtained with the SBS method. In the case of soft-tissue reconstruction, all models (2, 4, 5, 8, and 9) were similarly effective. They achieved the same values of TPR and TNR indices, equal to 95%. Detailed data on the structure of the models are presented in Table 3, whereas the confusion matrices of the most effective models for both types of reconstruction are presented in Figure 7.

## 4. Discussion

The quality of the built models should be assessed as high. In the case of bone reconstruction, four models (1, 2, 3, and 5) achieved the maximum accuracy for the test set (*ACC* = 92%); model no. 5 was characterized by the greatest sensitivity (*TPR* = 95%) at specificity *TNR* = 89%. In turn, for the soft-tissue reconstruction, the highest accuracy (*ACC* = 95%) was obtained for five models (2, 4, 5, 8, and 9). The remaining quality indices of these models, i.e., *TPR* and *TNR*, were equal to 95%. It should be emphasized that for both types of reconstruction, the majority of the models are characterized by relatively high validation accuracy. In turn, for the best models, the testing accuracy is only slightly lower than the validation accuracy. For bone reconstruction, this difference amounts only to 2%, and for soft-tissue reconstruction—1%. Such a situation indicates high quality, both of the models themselves, as well as the data used in the course of their training. The disadvantage of this method is that the extraction of the regions of interest was carried out manually. However, this problem requires separate investigations; therefore, it will be the topic of further research. The obtained results were compared with the results from the papers on a similar matter conducted by other authors (Table 4). Taking into account the total classification accuracy (*ACC*), it can be seen that the obtained result is one of the highest and is only 1.6% lower than the results achieved in [45,46], where it amounted to 96.6%. However, the majority of results presented in the table are much lower, and in the case of Andersen et al. [47], it amounts only to 71.3%. Other parameters also approximate the best results. While analyzing the data, it can be stated that the presented method stands out from similar investigations and exhibits high diagnostic potential.

**Figure 8:**
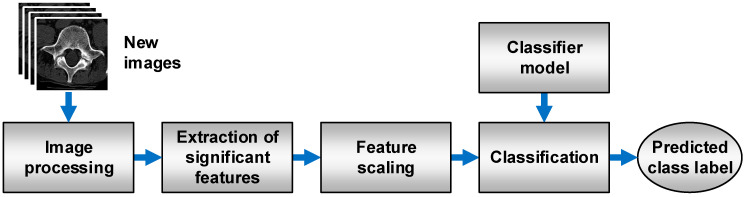
Process flow during the prediction of a class of new images.

**Figure 9:**
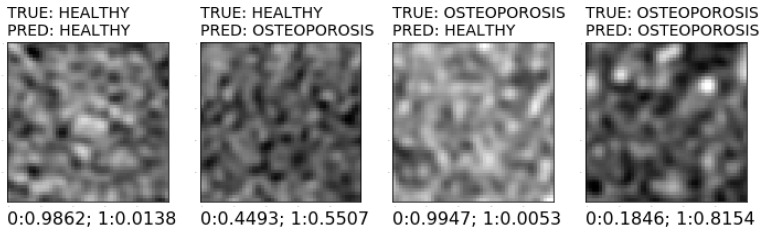
Various prediction results of sample images using soft tissue reconstruction.

Figure 8 shows the general scheme of a system for the prediction of new images with the constructed models. Initially, ROI extraction is conducted, and the 24-bit RGB type is changed to an 8-bit greyscale. The next stage is the determination of the feature descriptors, which were significant in the training process. The *K*-NN classifier (*K* = 1), considered the best for bone reconstruction, involves nine features that are obtained with the SBS method. In the case of soft-tissue reconstruction, a set of 17 or 18 features has to be determined in accordance with the applied classifier and selection method (Table 3). Then, the important features were scaled (standardization) with vectors of mean values and variance of features obtained during the scaling of training sets. Classification is the final step. On the basis of the classification model and the values of feature descriptors, the class is assigned to the analyzed image (prediction). Figure 9 shows prospective prediction results for exemplary images obtained through soft-tissue reconstruction. The presented results were obtained with a prototype system for diagnosing osteoporosis, which employs model no. 4 for prediction (Table 3).

## 5. Conclusions

The results of studies indicate that in the case of texture analysis, soft-tissue reconstruction is characterized by a higher classification accuracy (*ACC* = 95%) than bone reconstruction (*ACC* = 92%), which is usually employed for the spongy matter examination. Due to the atypical character of the examined tissue, exhibiting lower mineral density than in compact bone, the image from soft-tissue reconstruction contains a more accurate range of values pertaining to texture features. In that case, as many as five classifiers obtained the same maximum values of index quality (*ACC = TPR = TNR =* 95%). This justifies the usefulness of the conducted studies and indicates new possibilities stemming from an alternative approach to the analyzed tissue. The high efficiency of feature selection using the ANOVA, RELIEF, and RFE methods was observed as well. In combination with SVM and NuSVM classifiers, they enable achieving high classification accuracy. Studies showed that while designing a system for the automatic identification of osteoporotic bone fractures based on spinal CT, the application of soft-tissue reconstruction yields better results than bone reconstruction. It achieves 3% better classification accuracy than the traditional approach to the analysis of bone reconstruction images. Further investigations will focus on the development of an algorithm for automatic ROI extraction and testing the system utilizing a greater number of observations.

## Figures and Tables

**Table 1:** Results of hyperparametric optimization with the grid search method for the set of features obtained with the Fisher method (soft-tissue reconstruction). The criterion assumed for the selection of an optimal model for a given classification method involved achieving maximum validation accuracy with minimum number of training set features. The grey background was used to emphasize the model, which proved the best among the employed classification methods for the Fisher method. The meaning of parameters of individual classification models can be found in the scikit-learn library documentation [44].

Classification Method	Validation Accuracy	Optimal Features Number	Optimal Model Parameters
LDA	0.92	12	*solver* = ‘svd’
QDA	0.94	12	*tol* = 1 × 10^−5^
BAYES	0.91	11	*var_smoothing* = 0.1
SVM	0.96	16	*C* = 1.0, *gamma* = ‘scale’, *kernel* = ‘rbf’
NuSVM	0.96	16	*gamma* = ‘scale’, *kernel* = ‘rbf’, *nu* = 0.3
KNN	0.95	14	*n_neighbors* = 5
DT	0.91	10	*criterion* = ‘gini’, *max_depth* = 3
MLP	0.96	7	*activation* = ‘relu’, *alpha* = 0.1, *solver* = ‘lbfgs’, *max_iter* = 1000, *hidden_layer_sizes* = (3,)
RF	0.94	14	*max_depth* = 5, *n_estimators* = 20
GRAD	0.93	16	*loss* = ‘deviance’, *n_estimators* = 50
ADA	0.95	15	*n_estimators* = 50

**Table 2:** Basic information about optimal models for particular feature selection methods.

Model Number	Bone Reconstruction				Soft Tissue Reconstruction			
	Classification Method	Feature Selection Method	The Number of Features	Validation Accuracy (%)	Classification Method	Feature Selection Method	The Number of Features	Validation Accuracy (%)
1	NuSVM	FISHER	13	94	MLP	FISHER	7	96
2	RF	ANOVA	26	94	NuSVM	ANOVA	17	96
3	SVM	RELIEF	27	94	MLP	ANOVA	17	96
4	NuSVM	SFS	6	94	SVM	RELIEF	18	96
5	KNN	SBS	9	94	NuSVM	RELIEF	18	96
6	RF	SBS	9	94	KNN	SFS	5	96
7	MLP	RFE	18	95	KNN	SBS	5	96
8	ADA		9	93	SVM	RFE	18	96
9	LR		10	93	NuSVM	RFE	18	96
10	LGBM		3	89	ADA		8	95
11					LR		4	91
12					LGBM		2	88

**Table 3:** Information on the structure of models considered most effective for soft-tissue reconstruction.

Model Number	Classification Method	Feature Selection Method	The Number of Features	Model Parameters
2	NuSVM	ANOVA	17	*gamma* = ‘scale’, *kernel* = ‘rbf’, *nu* = 0.3
4	SVM	RELIEF	18	*C* = 1.0, *gamma* = ‘scale’, *kernel* = ‘rbf’
5	NuSVM	RELIEF	18	*gamma* = ‘scale’, *kernel* = ‘rbf’, *nu* = 0.3
8	SVM	RFE	18	*C* = 1.0, *gamma* = ‘scale’, *kernel* = ‘rbf’
9	NuSVM	RFE	18	*gamma* = ‘scale’, *kernel* = ‘rbf’, *nu* = 0.3

The meaning of the model parameters in Table 3: *gamma*—kernel coefficient; *kernel*—specifies the kernel type to be used in the algorithm; *nu*—an upper bound on the fraction of training errors and a lower bound of the fraction of support vectors; *C*—regularization parameter. The other model parameters, not listed in Table 3, take default values.

**Table 4:** Summary of results of similar bone texture analysis tests [48].

No. in Ref.	Texture Features	ROI Segmentation	Dataset	Classifier	*TPR*	*TNR*	*PPV*	*NPV*	*ACC*	F1-Score
Own results	Histogram, Gradient, Run length matrix, Cooccurrence, Autoregressive, Haar wavelet	Manual	50 cases & 50 controls	SVM NuSVM	95	95	-	-	95	-
[49]	power spectral density, triangular prism surface area and variation, box counting,	Manual	11 cases & 13 controls	K-NN	78	90	90	77	81	-
[50]	Wavelet Marginals-Haar	Calcaneal (Manual)	58 cases & 58 controls	SVM	62.1	65.5	64.3	63.3	63.8	63.2
[51]	1D LBP	Calcaneal (Manual)	39 cases & 41 controls	KNN	-	43.9	-	-	71.3	77.2
[47]	Fractal dimension, wavelet analysis, Gabor, LBP, DFT, DCT, Laws masks, edge histogram and GLCM	Calcaneal (Manual)	58 cases & 58 controls	RF	74.1	74.1	-	-	74.1	-
[52]	1D projection modeled as fractional Brownian motion	Calcaneal (Manual)	-	SVM	96.9	97.6	-	-	94.5	94.3
[45]	Fractional Brownian model and Rao geodesic distance	Calcaneal	348 cases & 348 controls	KNN	97.8	95.4	-	-	96.6	96.5
[46]	Histogram and GLCM and PCA analysis	Calcaneal (Manual)	87 cases & 87 controls	SVM	97.7	95.4	95.5	97.7	96.6	96.6
[53]	Anisotropic discrete dual-tree wavelet transform	Calcaneal (Manual)	87 cases & 87 controls	SVM	-	93.1	92.9	91.0	91.9	91.9
[54]	Wavelet decomposition and parametric circular models	Calcaneal (Manual)	87 cases & 87 controls	SVM	100	92.5	91.9	100	95.9	95.8
[55]	Oriental fractal analysis	Calcaneal (Manual)	87 cases & 87 controls	-	72.0	71.0	72.0	71.0	71.8	72.2
[56]	BMD, fractal, histomorphometric and skeletal measures	Distal radius	87 cases & 87 controls	SVM	79.0	66.0	-	-	-	-
[48]	Cortical, histogram, GLCM and MGM	Distal radius (Automated)	60 cases & 60 controls	SVM	86.7	65.0	71.2	83.0	75.8	78.2
[48]	Cortical and LLBP	Distal radius (Automated)	60 cases & 60 controls	SVM	88.3	66.7	72.6	85.1	77.5	79.7
[48]	Cortical and hLLBP	Distal radius (Automated)	60 cases & 60 controls	LR	81.7	76.7	77.8	80.7	79.2	79.7
[48]	Cortical and vLLBP	Distal radius (Automated)	60 cases & 60 controls	SVM	88.3	60.0	68.8	83.7	74.2	77.4

## Data Availability

Not applicable.
